# Successful Pregnancy Following Assisted Reproduction in Woman With Systemic Lupus Erythematosus and Hypertension: A Case Report

**DOI:** 10.1097/MD.0000000000001531

**Published:** 2015-09-18

**Authors:** José Fernando de Macedo, Gustavo Capinzaiki de Macedo, Luciana Aparecida Campos, Ovidiu Constantin Baltatu

**Affiliations:** From Reproferty Reproductive Medicine Clinic, Sao Jose dos Campos, São Paulo, Brazil (JFdM, GCdM, LAC); Center of Innovation, Technology and Education (CITE), Camilo Castelo Branco University, Sao Jose dos Campos, Sao Paulo, Brazil (JFdM, LAC, OCB); and Faculty of Medicine, Estácio de Sá University, Rio de Janeiro, Brazil (GCDM)

## Abstract

Patients with systemic lupus erythematosus have a poor prognosis of pregnancy, since it is associated with significant maternal and fetal morbidity, including spontaneous miscarriage, pre-eclampsia, intrauterine growth restriction, fetal death and pre-term delivery. We report a case with successful pregnancy in a patient with systemic lupus erythematosus and hypertension.

A 39-year-old nulliparous woman presented with systemic lupus erythematosus with antinuclear and antiphospholipid antibodies, hypertension and recurrent pregnancy loss presented for assisted reproduction. The patient responded well to enoxaparin and prednisone during both assisted reproduction and prenatal treatment.

This case report indicates that prescription of immunosuppressant and blood thinners can be safely recommended throughout the whole prenatal period in patients with systemic lupus erythematosus. Enoxaparin and prednisone may be prescribed concurrently during pregnancy.

## INTRODUCTION

Systemic lupus erythematosus (SLE) is an autoimmune, hypercoagulable state caused by antiphospholipid antibodies, being associated with antiphospholipid syndrome (APS). Patients with SLE have a poor prognosis of pregnancy,^1,2^ since it is associated with significant maternal and fetal morbidity, including spontaneous miscarriage, preeclampsia, intrauterine growth restriction, fetal death, and preterm delivery.^3,4^

Correct identification of women during pregnancy with SLE requires specific therapeutic care in order to improve fetal and maternal outcome.^5,6^ Pregnant patients with SLE and APS often require treatment with anticoagulant medication to reduce the risk of further episodes of thrombosis and improve the prognosis of pregnancy.^4^ The use of anticoagulants such as low-molecular weight heparin (LMWH) between weeks 15 and 34 of pregnancy in women with APS indicates good efficacy and safety.^5,7^

In vitro fertilization (IVF) and embryo transfer in women with SLE and APS may lead to embryonic loss or fetal death despite prednisone, hydroxychloroquine, and enoxaparin,^8^ stressing the importance of tight disease control and treatment. Assisted reproductive technology procedures including IVF do not appear to increase the risk of disease flare or thrombosis in patients with SLE and APS.^9^ Although assisted reproductive technologies can be successful in SLE and primary APS patients, rates of fetal and maternal complications are high.^10,11^ This is why we thought to report a case from our clinic with successful pregnancy in a patient with SLE and hypertension.

## METHODS

This is a case report of a patient at our reproductive medicine private practice. No Institutional Review Board approval was obtained. Our IRB designates a single-patient case report as not subject to IRB review because it does not meet the definition of human subjects research. The university ethics committee for human research has reviewed and approved the case report (CAAE 46103615.4.0000.5494). The patient has signed a consent form allowing disclosure of medical records.

## CASE REPORT

The patient is a 39-year-old nulliparous female who initially presented with infertility and right proximal tubal occlusion and endometriosis. Her history revealed that she was diagnosed 10 years ago with SLE according to the American College of Rheumatology criteria, including positive to antinuclear and anti-ribonucleoprotien antibodies tests.^12^ Also, the patient was diagnosed 3 years ago with hypertension. For SLE she was daily treated with low-dose corticosteroids, corticosteroid ointments, and hydroxychloroquine. For hypertension she was daily treated with enalapril 5 mg. The patient underwent 5 assisted reproductive technology cycles, of which 3 cycles were with transvaginal oocyte retrieval, 2 cycles for frozen–thawed embryo transfer, and 4 embryo transfer procedures. She continued on prednisone, hydroxychloroquine, and enalapril throughout the ovarian stimulation cycles.

During the assisted ovarian stimulation cycles treatments, enoxaparin sodium (Clexane, Sanofi-Aventis) 40 mg was administered to avoid thrombosis. Of the first 3 assisted ovarian stimulation cycles (Table 1), 2 were with short protocols using recombinant follicle-stimulating hormone (FSH, Pergoveris, Merck Serono, 225 IU SC daily) and GnRH antagonist (gonadotropin-releasing hormone, Cetrotide, Merck Serono, 0.25 mg SC daily) + ovulation triggering with choriogonadotropin alfa (recombinant human chorionic gonadotropin, r-hCG; Ovidrel, Merck Serono, 250 μg SC). The long protocol of the assisted ovarian stimulation cycle was with recombinant FSH (Pergoveris, Merck Serono, 225 IU SC daily), GnRH agonist (Lupron, Abbott, 5 IU SC) and luteinizing hormone (LH, Luveris, Merck Serono, 75 IU SC) + ovulation triggering with r-hCG. The induced plasma levels of progesterone and estradiol, and the resulting numbers of retrieved oocytes and embryos are presented in Table 1. No pregnancy was detected 14 days posttransfer after the first 2 assisted ovarian stimulation cycles.

**TABLE 1:**
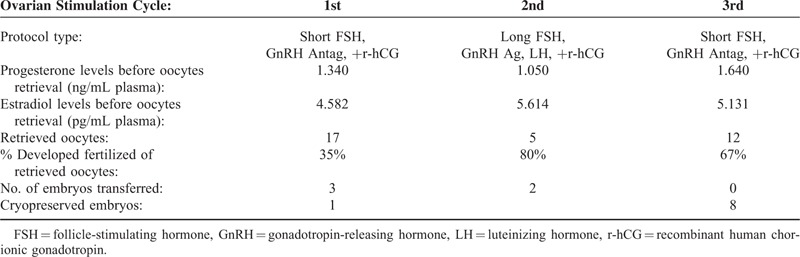
Ovarian Stimulation Cycles Outcomes

The fourth cycle was artificial cycle for frozen–thawed embryo transfer (AC-FET). The endometrial preparation was with estradiol (Primogyn, Bayer) PO in a dosage as follows: 2 mg days 1 to 6, 4 mg days 7 to 9, and 6 mg days 10 to 16. The endometrium was considered prepared when it was more than 7 mm thick on ultrasound, at which time vaginal progesterone (Crinone 8%, Merck Serono) thrice a day for 6 days was added to initiate secretory changes. Three preembryos were thawed and transferred. Pregnancy was detected by measuring plasma beta-hCG (β-hCG) (Table 2). Luteal support was achieved with estradiol (Primogyn, Bayer) PO 6 mg daily and vaginal progesterone (Crinone 8%, Merck Serono) thrice a day with pregnancy, and discontinued when the levels of β-hCG became negative for pregnancy.

**TABLE 2:**
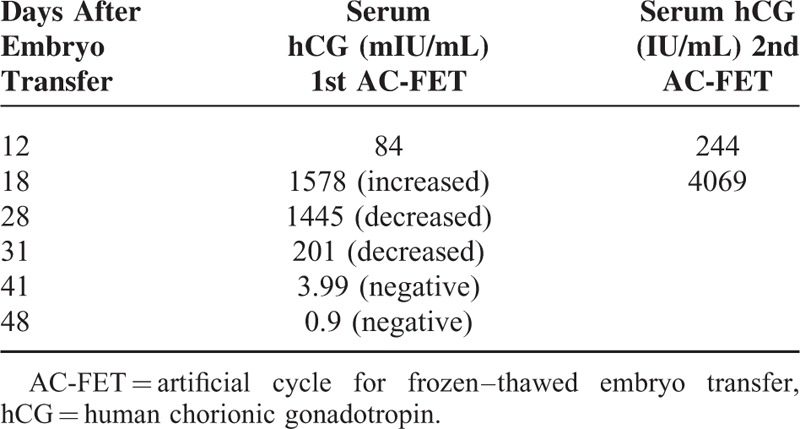
Pregnancy Tests With Serum hCG After AC-FET

The fifth cycle was artificial cycle AC-FET. Three preembryos were thawed, 2 survived and transferred. Pregnancy was detected by measuring plasma beta-hCG (Table 2). Luteal support was achieved with estradiol (Primogyn, Bayer) PO 6 mg daily and vaginal progesterone (Crinone 8%, Merck Serono) thrice a day with pregnancy, and discontinued at 12 weeks of gestation.

### Prenatal Follow-Up

Prenatal follow-up started with 6 weeks of gestation. Enoxaparin sodium (Clexane, Sanofi-Aventis) 60 mg SC daily was prescribed for the first 2 trimesters and 40 mg SC daily for the last trimester (Table 3). Prednisone (Meticorten, Schering Plough) 20 mg PO per day was prescribed until the third trimester when the dosage was decreased to 10 mg PO per day (Table 3). The antihypertensive treatment was changed from enalapril to aldomet (Methyldopa) 500 mg PO twice a day (Table 3). Pregnancy evolved without major complications. Toward the end of the pregnancy it appeared a cutaneous capillary fragility with cutaneous lesions. Also, a kidney stone was diagnosed during pregnancy and during lactation a kidney stone in each kidney and 1 in the bladder were found.

**TABLE 3:**
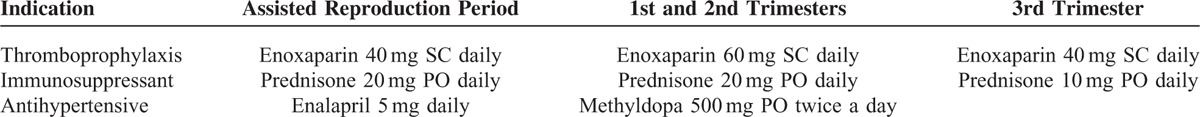
Prenatal Follow-Up

The patient delivered a healthy boy weighing 2.55 kg, 46 cm through C-section at 38th week of pregnancy.

## CONCLUSIONS

Our case report indicates that in assisted reproductive technology conception on patient with controlled SLE with hypertension may evolve without complications under adequate anticoagulant, immunosuppressant, and antihypertensive prescription.

Accumulating evidence from studies and case reports indicate risks of assisted reproductive technology in SLE patients, including ovarian hyperstimulation, repeated miscarriage, multifetal pregnancy, prematurity, and emotional distress.^2,9,10,13^ The current recommendations advise that SLE-affected woman achieve a stable remission of her renal disease and should have quiescent SLE for at least 6 months before conception.^9,14^ Current evidence indicates the relative safety of assisted reproductive technology procedures in patients with SLE.^9^ In a state of the art review, Ostensen et al^15^ conclude that pregnancies in women with APS remain a challenge, and better therapies for the obstetric APS are needed.

Immunosuppressants and anticoagulants are prescription drugs used to control lupus disease activity. Prednisone is the most commonly prescribed steroid for lupus and belongs to the category C of pregnancy risk (risk cannot be ruled out). This is why we decreased the prednisone dose to less than half of the reported average daily dose in a meta-analysis (27 mg).^16^ Immunomodulation with prednisone is considered as a promising treatment for recurrent pregnancy loss after assisted reproduction.^17^ We currently use concurrent immunomodulation with prednisone when there are at least 3 unsuccessful assisted reproductive technology cycles. In our protocol, prednisone starts from before embryo transfer and continues until pregnancy is established (14 days after embryo transfer). In our case, the patient had SLE with complicated hypertension controlled with enalapril already when she presented to the clinic. Since enalapril belongs to Category D (positive evidence of risk), we changed the treatment for the prenatal period to aldomet that belongs to Category B (no evidence of risk in humans).

Thromboprophylaxis using low-molecular-weight heparin (LMWH) in women with recurrent pregnancy loss after assisted reproduction and thrombophilia appear to increase implantation rate, pregnancy, and live birth rates.^18,19^ A Cochrane review suggests that LMWH may improve the live birth rate in women undergoing assisted reproduction, although evidence is highly debated.^20–22^ According to the RCOG Green-top Guideline No. 37a of the Royal College of Obstetricians and Gynaecologists (RCOG), women older than 35 years, or with SLE, with antiphospholipid antibodies are considered at risk and antenatal thromboprophylaxis throughout pregnancy is recommended.^23^ Also, these guidelines indicate “women with an IVF pregnancy and 3 other risk factors should be considered for thromboprophylaxis with LMWH starting in the first trimester.” Enoxaparin sodium is an LMWH that blocks factor Xa and factor IIa. According to FDA, enoxaparin sodium belongs to the category B of pregnancy risk (no evidence of risk in humans). In a case report, Joffe et al^24^ recommended that pregnant patients with APS should be considered candidates for full anticoagulation treatment throughout the entirety of gestation. We currently use concurrent thromboprophylaxis with low-dose aspirin (as a first choice) or LMWH (when assisted cycle is unsuccessful with low-dose aspirin) when there are at least 3 unsuccessful assisted reproductive technology cycles. In our protocol, an antithrombotic (low-dose aspirin or LMWH) starts from 3 days before embryo transfer and continues throughout the pregnancy.

Combined treatment of prednisone for immunosuppression and aspirin or LMWH as antithrombotic, starting before ovulation induction, may improve pregnancy rate in patients who have had repeated assisted reproductive failures.^25–27^ Our case indicates that concurrent immunosuppressant and antithrombotic treatment throughout the pregnancy is safe.

This case report indicates that prescription of immunosuppressant and blood thinners can be safely recommended throughout the whole prenatal period in patients with SLE. Enoxaparin and prednisone may be prescribed concurrently during pregnancy.
